# Loss anticipation and outcome during the *Monetary Incentive Delay Task*: a neuroimaging systematic review and meta-analysis

**DOI:** 10.7717/peerj.4749

**Published:** 2018-05-10

**Authors:** Jules R. Dugré, Alexandre Dumais, Nathalie Bitar, Stéphane Potvin

**Affiliations:** 1 Department of Psychiatry, University of Montreal, Montreal, Quebec, Canada; 2 Centre de recherche de l’Institut Universitaire en Santé Mentale de Montréal, Montreal, Quebec, Canada; 3 Institut Philippe-Pinel de Montréal, Montreal, Quebec, Canada

**Keywords:** Monetary Incentive Delay Task, Meta-analysis, fMRI, Punishment, Loss avoidance

## Abstract

**Background:**

Reward seeking and avoidance of punishment are key motivational processes. Brain-imaging studies often use the *Monetary Incentive Delay Task* (MIDT) to evaluate motivational processes involved in maladaptive behavior. Although the bulk of research has been done on the MIDT reward events, little is known about the neural basis of avoidance of punishment. Therefore, we conducted a meta-analysis of brain activations during anticipation and receipt of monetary losses in healthy controls.

**Methods:**

All functional neuro-imaging studies using the MIDT in healthy controls were retrieved using PubMed, Google Scholar & EMBASE databases. Functional neuro-imaging data was analyzed using the Seed-based d Mapping Software.

**Results:**

Thirty-five studies met the inclusion criteria, comprising 699 healthy adults. In both anticipation and loss outcome phases, participants showed large and robust activations in the bilateral striatum, (anterior) insula, and anterior cingulate gyrus relatively to Loss > Neutral contrast. Although relatively similar activation patterns were observed during the two event types, they differed in the pattern of prefrontal activations: ventro-lateral prefrontal activations were observed during loss anticipation, while medial prefrontal activations were observed during loss receipt.

**Discussion:**

Considering that previous meta-analyses highlighted activations in the medial prefrontal cortex/anterior cingulate cortex, the anterior insula and the ventral striatum, the current meta-analysis highlighted the potential specificity of the ventro-lateral prefrontal regions, the median cingulate cortex and the amygdala in the loss events. Future studies can rely on these latter results to examine the neural correlates of loss processing in psychiatric populations characterized by harm avoidance or insensitivity to punishment.

## Introduction

Reward seeking and avoidance of punishment both play a key role in human motivation (Navratilova & Porreca, 2014). Both components of motivation, when expressed in excess or insufficiency, can be associated with maladaptive behavior. Indeed, several studies have shown that individuals with major depressive disorder and schizophrenia both lack motivation for rewards (Takamura et al., 2017; Whitton, Treadway & Pizzagalli, 2015), whereas individuals with substance use disorders have uncontrolled motivation for substance seeking but decreased motivation for alternative natural rewards (Baker et al., 2017). There is also evidence individuals with anxiety disorders are characterized by harm avoidance (Ottenbreit, Dobson & Quigley, 2014; Wright, Lebell & Carleton, 2016), whereas individuals with antisocial behavior tend to be insensitive to punishment (Byrd, Loeber & Pardini, 2014; Loney et al., 2003).

In view of the importance of reward seeking and avoidance of punishment to human behavior and maladaptive behavior, diverse cognitive tasks have been developed to study both processes in humans; the most employed being the *Monetary Incentive Delay Task* (MIDT) (Knutson & Greer, 2008). Although several versions of the task exist, they similarly subdivide events of reward anticipation and receipt, with relatively fewer versions also comprising events of loss anticipation and outcome (Balodis & Potenza, 2015; Patel et al., 2013). The MIDT and its several variants have been very useful in studying the neurobiological mechanisms in reward processing. Knutson & Greer (2008) performed a neuro-imaging meta-analysis which showed that healthy participants recruit the bilateral nucleus accumbens (NAC), thalamus, the right (anterior) insula and the medial frontal gyrus during reward *anticipation* (*n* = 20 studies), while they activate the bilateral NAC, the right caudate nucleus, the left amygdala and the right sub-callosal gyrus during reward receipt (*n* = 12 studies). Since then, a much larger meta-analysis has been performed by Liu et al. (2011), which did not restrict the inclusion of studies to those using the MIDT specifically. In this meta-analysis, comprising of a total of 142 neuro-imaging studies, it was shown that the reward *anticipation* is associated, in healthy volunteers, with activations in the bilateral NAC, bilateral (anterior) insula, bilateral (dorsal) anterior cingulate cortex (ACC) and the left medial orbito-frontal cortex (OFC), while reward *receipt* is associated with similar activations in the bilateral NAC, insula, medial OFC, the right amygdala and thalamus. Taken together, the results of these meta-analytic studies highlight activations during reward processing in dopamine-rich brain regions (e.g., NAC, insula, ACC and medial OFC), a finding consistent with the vast pre-clinical literature showing that meso-cortico-limbic dopaminergic neurons are critically involved in the processing of both drug and natural rewards (Lammel et al., 2011; Pignatelli & Bonci, 2015; Pitchers et al., 2010).

The growing understanding of the neurobiological bases of reward processing has fueled research on motivational alterations in psychiatric disorders. Thus far, several studies and meta-analyses have highlighted reduced activations in the ventral striatum (VS) during reward anticipation and receipt in schizophrenia (Radua et al., 2015); reduced VS activations during reward anticipation and increased VS activations during reward receipt in addiction (Luijten et al., 2017); as well as decreased sub-cortical and limbic regions and increased cortical responses during reward processing in major depressive disorder (Zhang et al., 2013). Likewise, blunted VS responses have been observed in large-scale studies of adolescents at risk of addictive behaviors (Büchel et al., 2017; Jia et al., 2016). Comparatively, it is striking to observe that little attention has been paid to the study of loss anticipation and receipt events in both healthy and psychiatric populations. This means that at the present, the neural map of activations associated with loss events is unknown, although one of the lead theories of antisocial behavior proposes that individuals will not readily obey to the law if they are insensitive to punishment (Byrd, Loeber & Pardini, 2014; Crowley et al., 2010). In the meta-analysis of Liu et al. (2011) involving healthy participants, no specific analysis was performed on loss events (anticipation or receipt). In the meta-analysis of Knutson & Greer (2008), also involving healthy participants, a sub-analysis on 12 studies revealed activations of the right caudate nucleus, the left putamen, the left thalamus and the left (dorsal) insula during loss *anticipation*, while activations of the superior temporal gyrus was observed during loss *receipt*. The reliability of the latter result is especially uncertain, given that it was based on the pooling of only six studies. Moreover, about a third of the studies included in this meta-analysis were studies using predefined regions-of-interest (ROIs) rather than performing whole-brain analyses, and this methodological choice may have biased results. In this context, some authors have noticed that the regions and pathways that are differently activated in healthy participants during reward versus loss events remain unknown (Knutson & Greer, 2008; Lutz & Widmer, 2014). In theory, it has been proposed that rewarding events may elicit stronger activations in the medial prefrontal cortex (medial OFC and ventral ACC) and VS (Dillon et al., 2008; Schlagenhauf et al., 2009), given that these regions are well known core regions of the brain reward system (Lammel et al., 2011; Pignatelli & Bonci, 2015; Pitchers et al., 2010). Conversely, some authors have proposed that stronger activations may occur during loss events in the amygdala (Hahn et al., 2010; Lutz & Widmer, 2014), a region critically involved in threat detection (LeDoux, 2014), as well as the hippocampus (Hahn et al., 2010), which plays an important role in memory retrieval of negative emotions (Fossati, 2012). On the other hand, some authors have argued that certain brain regions may be involved in the processing of both rewarding and loss events. For instance, Hahn et al. (2010) have postulated that the dorsal ACC could be activated during both reward and loss *anticipation* (regardless of valence), since the anticipatory phase is characterized by heightened arousal and increased attention. Finally, Wu et al. (2014) noticed that the (anterior) insula is likely to play a role in the processing of both rewarding and loss events, since this brain region responds to affective stimuli of positive and negative valence (Fusar-Poli et al., 2009; Liu et al., 2011; Palermo et al., 2015). These hypotheses need to be further investigated.

In view of our poor understanding of the neurobiological bases of punishment processing, we sought to perform a functional neuro-imaging meta-analysis of loss events (anticipation and receipt) in healthy participants. Analyses were restricted to the studies using MIDT in order to reduce task heterogeneity.

## Method

### Selection procedures

#### Search strategies

A systematic search strategy was employed to identify relevant studies for the present meta-analysis. The literature search was performed by two researchers (JD, SP), independently, with the use of PubMed, Google Scholar and EMBASE search engines, up to September 2017. The following search terms were used: “MID” (monetary incentive delay) AND “loss” or “loss-avoidance” or “punishment” AND “fMRI” (functional magnetic resonance imaging). Also, a cross-referencing method was used by manually examining reference lists of the articles included in the meta-analysis.

#### Selection criteria

Studies were included if they met the following criteria: (1) were reported in an original paper from a peer-reviewed journal, (2) had involved healthy subjects (i.e., no psychiatric or organic disorders reported) as a primary or control group, (3) had employed the *Monetary Incentive Delay Task* (MIDT) (Knutson et al., 2000) or a modified version of the MIDT (Bjork et al., 2004), (4) had included punishment cues (loss or receipt) in the task and reported brain map activations for this component. Studies were reviewed by two researchers (JD, SP) and inclusion criteria were evaluated by consensus. To achieve a high reporting standard, we followed the “*Preferred Reporting Items for Systematic Reviews and Meta-Analyses*” (PRISMA) guidelines (for more information, see Table S1) (Moher et al., 2009).

#### Recorded variables

The variables included in the present meta-analysis, for each article, were: sample size, mean age of participants, magnet intensity and repetition time (TR) of functional volumes. Also, recent research has suggested that the use of full width at half maximum (FWHM) of the smoothing kernel (Sacchet & Knutson, 2013) are leading to heterogeneous results in neuro-imaging studies. Therefore, these variables were also recorded in the meta-analysis (see Table 1 for complete data reports).

#### Meta-analysis

The meta-analysis was performed by using the *Effect-size Seed-based d Mapping* (formerly *Signed Differential Mapping*) (ES-SDM) (Radua et al., 2012a; Radua et al., 2012b). This method is based on the use of peak coordinates to recreate, for each study, an effect-size map of contrast results. A standard random-effects variance weighted meta-analysis for each voxel is then executed. Default ES-SDM kernel size and thresholds were used (FWHM = 20 mm, voxel *P* = 0.005, peak height *Z* = 1, cluster extent = 10 voxels) (Radua et al., 2012a; Radua et al., 2012b).

**Table 1:** Description of the studies included in the meta-analysis (*n* = 35).

First author (year)	*N*	Type?	Loss outcome	Mean age	Software	Tesla	FWHM	TR	Incentive magnitude	Percentage of winning
Balodis et al. (2012)	14	ROI	X	37.1	spm5	3	6	1500	1$ & 5$	66.6%
Bayer, Bandurski & Sommer (2013)	23	ROI	–	26	spm8	3	8	2630	€0.20, €3	50.0%
Beck et al. (2009)	19	WB + ROI	X	41.68	spm5	1.5	8	1870	€0.10, €0.60, €3	66.6%
Bjork et al. (2004)	12	ROI	X	23.8	AFNI	3	2	2000	0.20$, 1$, 5$	66.6%
Bjork, Smith & Hommer (2008)	23	ROI	–	32	AFNI	3	8	2000	0.50$, 5$	66.6%
Bjork et al. (2010)	24	ROI	X	29.3	AFNI	3	8	1000	0.50$, 5$	67.0%
Bustamante et al. (2014)	18	WB	–	37.44	spm8	1.5	8	2000	€0.20, €3	75.0%
Cho et al. (2013)	30	ROI	–	28.8	spm8	3	8	2500	0.20$, 1$, 5$	66.6%
Cooper et al. (2009)	12	WB	X	NA	spm2	1.5	4	2000	0.05$, 5$	66.6%
Dillon et al. (2008)	8	ROI	X	28.13	AFNI	1.5	6	2500	Range (1.81 to 2.19$)	50.0%
Enzi et al. (2012)	15	ROI	–	34.7	spm5	1.5	8	1900	€0.10, €0.60, €3	66.6%
Hahn et al. (2010)	45	WB	–	29.1	spm5	1.5	NA	2000	€0.05, €1	67.0%
Herbort et al. (2016)	23	WB	–	25.78	spm8	3	8	2000	€0.50, €10	66.6%
Juckel et al. (2006)	10	WB + ROI	–	31.7	spm2	1.5	8	1900	€0.10, €0.60, €3	66.6%
Juckel et al. (2012)	13	WB	–	25.69	spm5	1.5	8	1987	NA	NA
Kaufmann et al. (2013)	19	WB	–	34.9	spm8	1.5	8	1870	€0.10, €0.60, €3	66.6%
Kirk, Brown & Downar (2014)	44	ROI	X	36.5	spm8	3	8	2000	1$, 5$	55.0%
Knutson et al. (2001)	8	ROI	–	31	AFNI	1.5	4	2000	0.20$, 5$	66.6%
Knutson et al. (2003)	12	ROI	–	31	AFNI	1.5	4	2000	0.20$, 1$, 5$	66.6%
Knutson & Greer (2008)	12	WB	X	28.67	AFNI	1.5	4	2000	0.20$, 1$, 5$	66.6%
Kocsel et al. (2017)	37	WB	X	25.92	spm12	3	8	2500	Range (1.76 to €2.12)	NA
Pfabigan et al. (2014)	25	ROI	–	23.8	spm8	3	8	1800	€2.00	50.0%
Romanczuk-Seiferth et al. (2015)	17	WB	X	37.41	spm8	3	8	2500	€1.00	67.0%
Samanez-Larkin et al. (2007)	12	WB	X	23.75	AFNI	1.5	4	2000	0.50$, 5$	66.6%
Samanez-Larkin et al. (2007)	12	WB	X	72.92	AFNI	1.5	4	2000	0.50$, 5$	66.6%
Schlagenhauf et al. (2008)	10	WB	–	31.8	spm2	1.5	4	1900	€0.10, €0.60, €3	66.6%
Schlagenhauf et al. (2009)	15	WB + ROI	X	30.1	spm5	1.5	8	1987	€0.10, €0.60, €3	66.6%
Stoy et al. (2011)	12	WB + ROI	–	28.08	spm5	1.5	8	1900	€0.10, €0.60, €3	66.6%
Stoy et al. (2012)	15	WB	–	39.5	spm5	1.5	8	1900	€0.10, €0.60, €3	66.6%
Treadway, Buckholtz & Zald (2013)	38	WB	X	22	spm5	3	6	2000	0.20$, 1$, 5$	66.6%
Ubl et al. (2015)	28	WB + ROI	X	43.96	spm5	3	6	2700	€0.20, €2	65.0%
Van Duin et al. (2016)	12	WB	–	29	spm8	3	8	2000	0.20$, 1$, 5$	NA
Wrase et al. (2007a)	14	WB	–	39.9	spm2	1.5	6	1870	€0.10, €0.60, €3	67.0%
Wrase et al. (2007b)	16	ROI	–	39.94	spm2	1.5	4	1800	€0.10, €0.60, €3	67.0%
Wu et al. (2014)	52	WB	X	50	AFNI	1.5	4	2000	0.50$, 5$	66.6%

**Note:**

WB Whole-Brain ROI Region Of Interest AFNI Analysis of Functional NeuroImages SPM Statistical Parametric Mapping Tesla Scanner Magnet Intensity FWHM Full Width at Half Maximum Smoothing Kernel level TR Repetition Time

Also, robustness of the significant results was assessed by means of exploration of the residual heterogeneity, jack-knife and subgroup analyses. Furthermore, we investigated if the findings had been driven by a small subset of studies or studies including small samples. Publication bias was assessed by examining Egger’s tests (Egger et al., 1997) for asymmetry of the funnel plots (Sterne et al., 2011). Jack-knife sensitivity analyses consisted of repeating the meta-analysis iteratively by removing one study at a time to assess the replicability of the results (Radua et al., 2012a; Radua et al., 2012b). Subgroup analyses were conducted on magnet intensity (1.5 tesla *versus* 3 tesla) and the smoothing kernel used (FWHM = 4 *versus* FWHM = 8). Finally, a meta-regression was performed on mean age of participants and TR across studies. Following previous meta-analyses, we decreased the probability threshold to minimize the detection of spurious results (please refer to Radua et al., 2012a; Radua et al., 2012b; Radua & Mataix-Cols, 2009 for further details on robustness analyses).

## Results

### Number of studies found

**Figure 1:**
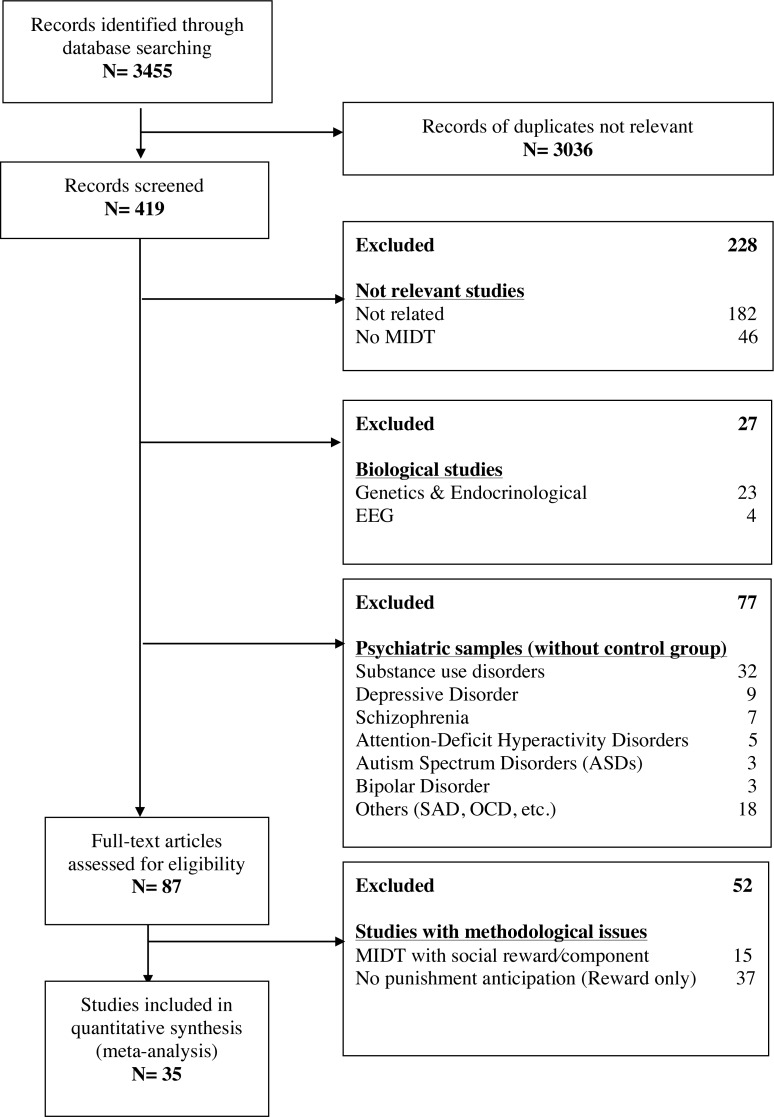
Flow chart of the studies included in the meta-analysis.

Thirty-five studies met inclusion criteria for this loss anticipation meta-analysis (see Fig. 1 for the flow chart). More specifically, we included 699 healthy individuals (mean TR 2014.69 ms, range 1,000–2,700 ms; mean age 33.28, range 22–72.92). Twenty-three studies used a whole-brain analysis and twelve used predefined regions-of-interest in their statistical analyses. Also, a large majority of studies used a MID task with ≥65% chance of successful trial (*n* = 28, 80%). All studies reported similar monetary incentive per stimulus (largest inventive = 5$ or 3 euros, except for one study reporting 10$). Of these 35 studies, 16 also reported loss outcome brain activations in their results comprising 356 healthy subjects (mean TR 2034.81ms, range 1,000–2,700 ms; mean age 35.42, range 22–72.92). Ten studies examined whole brain activations and six used predefined region-of-interest. For more details of the included studies, please see Table 1.

### Brain responses during loss anticipation

**Figure 2:**
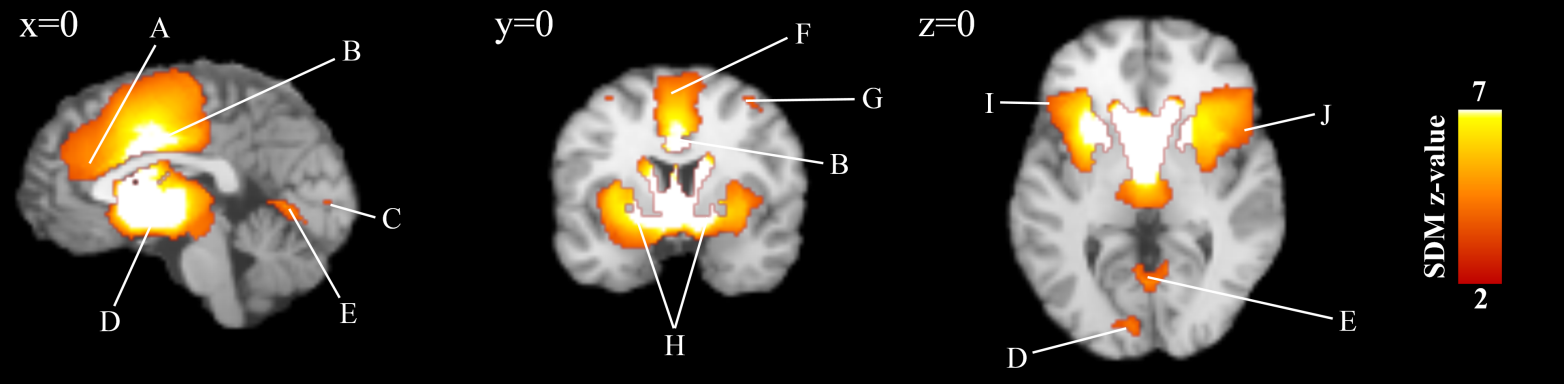
Overlay of brain areas activated in loss anticipation events (*x* = 0, *y* = 0, *z* = 0). These blobs were generated using the SDM *p*-value threshold of *p* = 0.005 derived from the original analysis. SDM = Seed-Based d Mapping. (A) Anterior Cingulate Cortex. (B) Median Cingulate Cortex. (C) Lingual Gyrus. (D) Striatum. (E) Vermis. (F) Precentral Gyrus. (G) Medial Frontal Gyrus. (H) Lentiform Nucleus. (I) Inferior Frontal Gyrus. (J) Insula.

Subjects showed a large and robust bilateral striato-insular activation cluster (*Z* = 6.86, Cluster size = 10,623, *p* < 0.001) that includes the bilateral anterior insula, the putamen, the thalamus, the caudate nucleus and the amygdala, as well as the bilateral ventro-lateral sub-regions. Significant increased activations were also observed in the bilateral median cingulate gyri (*Z* = 5.69, Cluster size = 4,845, *p* < 0.001), the left precentral gyrus (*Z* = 3.95, Cluster size = 846, *p* < 0.001), the bilateral cerebellum hemispheric lobule VI (Left: *Z* = 3.50, Cluster size = 551, *p* < 0.001, Right: *Z* = 3.13, Cluster size = 144, *p* = 0.002), the bilateral lingual gyrus (Left: *Z* = 3.42, Cluster size = 129, *p* < 0.001; Right: *Z* = 3.70, Cluster size = 182, *p* < 0.001) as well as the right middle frontal gyrus (*Z* = 3.17, Cluster size = 130, *p* = 0.002) (Table 2; Fig. 2). The analyses of robustness showed that regions were highly replicable with the exception of the right cerebellum hemispheric lobules VI, the left lingual gyrus and the right middle frontal gyrus, which were found in only 68.6%, 74.3% and 68.6% of the 35 studies (see Table S3).

Furthermore, funnel plots revealed that only the peak activation of the striato-insular cluster may have been driven by small or noisy studies. In fact, significant publication bias was observed in the peak of the striato-insular cluster (*x* = 12, *y* =  − 4, *z* = 0), as shown by the Egger’s test result (Bias: 3.24, *t*: 3.93, *df*: 33, *p* < 0.001). However, every study included in the meta-analysis reported activations in the striato-insular cluster. Considering the large size of this cluster and its very large effect size, the publication bias found in the main peak is unlikely to reduce the robustness and validity of the results. In fact, results within this cluster comprised an outlier study (e.g., Kaufmann et al., 2013). When removing the peak activations results within the striato-insular cluster from this outlier study, we still observed highly similar results (Peak at *x* =  − 2, *y* = 4, *z* =  − 2; *Z* = 7.11, Cluster size = 10,030, *p* < 0.001) but no publication bias (Bias: 1.03, *t*: 1.34, *df*: 33, *p* = 0.191) (see Table S2 and Fig. S1 for Funnel Plot). No publications bias was observed for the bilateral median cingulate gyri (Bias: 0.76, *t*: 1.24, *df*: 33, *p*: 0.225), the left precentral gyrus (Bias: −0.46, *t*: − 0.68, *df*: 33, *p*:0.503), the bilateral cerebellum hemispheric lobule VI (Left: Bias: 0.26, *t*: 0.39, *df*: 33, *p*: 0.700; Right: Bias: 1.13, *t*: 1.29, *df*: 33, *p*: 0.207), the bilateral lingual gyrus (Left: Bias: 1.24, *t*: 1.85, *df*: 33, *p*: 0.074; Right: Bias: −0.53, t: −0.76, *df*: 33, *p*: 0.450) as well as the right middle frontal gyrus (Bias: 0.14, *t*: 0.21, *df*: 33, *p*: 0.836).

**Table 2:** Increased activations during anticipation of monetary loss (all studies; *n* = 35).

	*MNI* Coordinates	SDM *z*-value^a^	*P* Value^b^	No. of voxels^c^	Breakdown (No. of voxels)^d^
Left lenticular nucleus	−12,4,0	6.863	∼0	10,623	Bilateral insula (1,635)
					Bilateral anterior thalamic projections (1,253)
					Bilateral striatum (1,243)
					Bilateral putamen (755)
					Bilateral caudate nucleus (581)
					Bilateral thalamus (563)
					Bilateral BA 47 (374)
					Bilateral BA 25 (331)
					Bilateral BA 45 (239)
					Corpus callosum (230)
					Bilateral BA 11 (217)
					Bilateral BA 34 (130)
					Bilateral amygdala (129)
					Anterior commissure (112)
Left median cingulate gyri	0,0,32	5.694	∼0	4,845	Bilateral median cingulate gyri (1,917)
					Bilateral supplementary motor area (1548)
					Bilateral Anterior cingulate gyri (655)
					Corpus callosum (327)
					Bilateral superior frontal gyrus, medial (255)
Left precentral gyrus	−34,−24,58	3.945	0.000026	846	L precentral gyrus (473)
					L postcentral gyrus (319)
					Corpus callosum (33)
Left cerebellum, lobule VI	−20, −70, −14	3.503	0.00037	551	L hemispheric lobule VI (366)
					L crus I (116)
Right Lingual Gyrus	0, −66,0	3.704	0.00012	182	R Lingual Gyrus (60)
					Cerebellum, vermic lobule IV/V (30)
Right cerebellum, lobule VI	16, −62, −16	3.134	0.0024	144	R hemispheric lobule VI (108)
					R lingual gyrus (13)
Left lingual gyrus	−8, −86,2	3.416	0.00059	129	L BA 17 (49)
					Corpus callosum (31)
Right middle frontal gyrus	36,0,54	3.168	0.002	130	R BA 6 (124)

**Notes.**

BA Brodmann Area SDM Seed-based d Mapping

a Voxel probability threshold: *p* = 0.005.

b Peak height threshold: *z* = 1.

c Cluster extent threshold: 100 voxels.

d Regions with less than 10 voxels are not reported in the cluster breakdown.

We also found significant residual heterogeneity between studies in activations during loss anticipation (*τ* = 0.06, *Q* = 66.1, *df* = 34, *p* = 0.0008). To better understand this heterogeneity, we performed subgroup analyses. First, no significant difference was observed between whole-brain studies *versus* region-of-interest studies. Second, magnet intensity subgroup analysis yielded significant results. In fact, studies using a 3 Tesla magnet reported more frequently increased activations in the left cerebellum (hemispheric lobule VI) (*Z* = 2.86, Cluster size = 1,295, *p* < 0.001) and the left thalamus (*Z* = 3.58, Cluster size = 350, *p* < 0.001) while studies using a 1.5 Tesla magnet reported more frequently increased activations in the bilateral left inferior frontal gyrus (Left: *Z* = 2.07, Cluster size = 297, *p* < 0.001; Right: *Z* = 2.24, Cluster size = 1,062, *p* < 0.001) (see Table S4). Third, comparisons between the kernel density employed in smoothing parameterizations showed that studies using a 8 mm FWHM reported more increased activations in the left lingual gyrus ( *Z* = 2.25, Cluster size = 512, *p* < 0.001) and the left thalamus (*Z* = 2.14, Cluster size = 120, *p* < 0.001) (see Table S4) while 4 mm FWHM yielded in increased activations in the right insula (*Z* = 3.82, Cluster size = 186, *p* < 0.001). Finally, the meta-regression revealed significant age and TR effects. In fact, studies with older participants reported more increased activations in the left inferior frontal gyrus (opercular part) (*Z* = 2.62, Cluster size=190, *p* = 0.0011) as well as the left median/posterior cingulate gyrus (*Z* = 2.83, Cluster size = 182, *p* < 0.001) while studies with younger participants reported more increased activations in the left anterior cingulate gyrus (*Z* = 2.25, Cluster size = 941, *p* < 0.001), right olfactory cortex (*Z* = 2.75, Cluster size=611, *p* < 0.001), left thalamus (*Z* = 2.59, Cluster size=550, *p* < 0.001) as well as the right lingual gyrus (*Z* = 2.09, Cluster size = 182, *p* < 0.001) (see Table S6). Finally, regarding the functional TR, shortest TR was significantly associated with increased activations in the lingual gyrus (*Z* = 2.99, Cluster size = 461, *p* < 0.001) as well as the right cerebellum (vermic lobule VI) (*Z* = 2.60, Cluster size = 297, *p* < 0.001) (see Table S7).

### Brain responses during loss receipt/outcome

**Figure 3:**
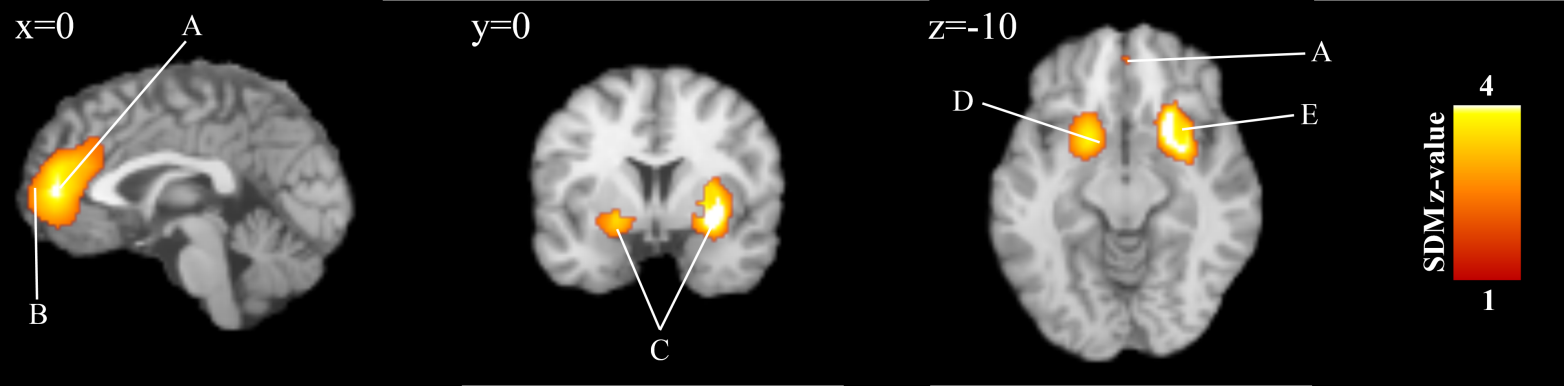
Overlay of brain areas activated in reception of loss events (outcome)(*x* = 0, *y* = 0, *z* =  − 10) These blobs were generated using the SDM *p*-value threshold of *p* = 0.005 derived from the original analysis. SDM = Seed-Based d Mapping. (A) Anterior Cingulate Cortex. (B) mFG = Medial Frontal Gyrus. (C) Lentiform Nucleus. (D) Globus Pallidus. (E) Claustrum.

Subjects also showed increased activations in a bilateral striato-insular cluster (Left: *Z* = 2.92, Cluster size = 567, *p* < 0.001; Right: *Z* = 3.39, Cluster size = 1,475, *p* < 0.001) that includes the putamen, the anterior insula and the amygdala (Table 3; Fig. 3). We also observed significant increased activations in the bilateral anterior cingulate/paracingulate gyri (encompassing the medial PFC) (*Z* = 3.14, Cluster size = 1,625, *p* < 0.001) during the loss outcome. These results were highly replicable as shown by the jack-knife analysis (See Table S8). No publication bias was observed for the bilateral striato-insular cluster (Left: Bias: 0.94, *t*: 1.11, *df*: 14, *p*: 0.285; Right: Bias: −0.06, *t*: −0.07, *df*: 14, *p*: 0.946), the bilateral anterior cingulate/paracingulate gyri (Bias: 0.49, *t*: 0.61, *df*: 14, *p*: 0.550) suggesting that these regions were not driven by few small or noisy studies. Finally, no significant residual heterogeneity was observed between studies (*τ* = 0.04, *Q* = 22.4, *df* = 15, *p* = 0.098).

**Table 3:** Increased activations during monetary loss (outcome) (*n* = 16).

	*MNI* Coordinates	SDM *z*-value^a^	*P* Value^b^	No. of voxels^c^	Breakdown (No. of voxels)^d^
Right striatum (putamen)	22,16,−8	3.389	0.000005	1,475	R BA 48 (775)
					R striatum (301)
					R Insula (94)
					R inferior network (79)
					R BA 47 (45)
					R putamen (27)
					R amygdala (20)
Left anterior cingulate gyri	−2,48,8	3.138	0.000015	1,625	Bilateral anterior cingulate gyri (1,060)
					Bilateral superior frontal gyrus (510)
Left striatum (putamen)	−18,6,−8	2.924	0.000026	567	L striatum (208)
					L putamen (118)
					L BA 48 (89)
					L BA 25 (32)
					L amygdala (28)
					L BA 11 (11)

**Notes.**

BA Brodmann Area SDM Seed-based d Mapping

a Voxel probability threshold: *p* = 0.005.

b Peak height threshold: *z* = 1.

c Cluster extent threshold: 100 voxels.

d Regions with less than 10 voxels are not reported in the cluster breakdown.

No subgroup analyses were performed in order to avoid abnormal cluster activations resulting from the small number of studies having reported the loss outcome results (*n* = 16).

## Discussion

Compared to the vast neuro-imaging literature on the neural mechanisms involved in reward processing (anticipation and outcome), little attention has been paid to how humans process punishments at the brain level. Here, we performed a neuro-imaging meta-analysis of loss events during the MIDT in healthy volunteers, using the seed-based d mapping approach. The meta-analysis showed that during loss *anticipation*, participants activated the bilateral insula, bilateral caudate nucleus and putamen, bilateral amygdala, bilateral ventro-lateral prefrontal areas, as well as the median and anterior cingulate gyri, the left pre- and post-central gyri, and the left cerebellum. Activations were also observed, during loss *anticipation*, in the bilateral lingual gyrus, the right cerebellum and middle frontal gyrus, but these results were less robust, as revealed by the jacknife sensitivity analysis. Results were found to be influenced by the mean age of participants, the TR, the scanner magnet intensity and the smoothing kernel level. Relative to loss anticipation, loss *outcome* was associated with activations in similar brain regions, though the cluster size was smaller in the case of loss outcome. That is, the loss outcome event was associated, in healthy participants, with activations in the bilateral striatum (putamen), bilateral amygdala, right ventro-lateral prefrontal cortex, and ACC (encompassing the medial PFC). Most of these regions are related to the emotional salience network that has been identified using rest- and task-based functional connectivity analyses (Menon & Uddin, 2010). Importantly, the pattern of activations between loss *anticipation* and loss *outcome* differed in that the former was associated with activations of *ventro-lateral* prefrontal regions, whereas the latter was associated with activations of the *medial* PFC.

The finding that the loss events recruit activations in the ACC, anterior insula and striatum is consistent with a large literature showing that these brain regions are critically involved in the affective responding to a whole range of stimuli having a negative valence, such as faces expressing fear or anger, images depicting social conflicts, sad music or nociceptive stimuli (Fusar-Poli et al., 2009; Groenewold et al., 2013; Koelsch, 2010; Palermo et al., 2015). It must be noticed that in previous meta-analyses on reward anticipation and receipt, activations in the (ventral) striatum, anterior insula and ACC were also observed, meaning that the regions are commonly activated by both types of reinforcers, regardless of their valence, and do not differentiate between them. As such, this result is unsurprising, as the challenge of establishing the pattern of activity preferentially associated with reward and punishment has been noticed by several authors (Byrd, Loeber & Pardini, 2014; Liu et al., 2011; Lutz & Widmer, 2014). Before concluding that the ACC, anterior insula and striatum lack specificity for the valence of reinforcers, it is important to point out, however, that the similarities in activations between the processing of reward and punishment may stem from a bias in the selection of ROIs. Indeed, most studies included in the meta-analysis which performed ROI analyses used regions previously found (by them or others) to be activated during reward outcomes. Obviously, this selection of ROIs may have introduced a bias towards finding activations in reward-related brain regions during punishment. Because we were conscious of this potential bias at the beginning, we performed a sub-analysis comparing ROI to whole-brain analyses. Importantly, we found no significant differences in activations between studies that perform whole-brain analyses versus those who used a priori defined ROIs. This strongly suggests that the similarities between our results and results of previous meta-analyses on reward processing are unlikely to be explained by a biased selection of ROIs.

Perhaps more interestingly, the meta-analysis produced results suggesting that there might be subtle differences between the neural processing of reward and punishment. First, whereas the previous meta-analysis of Liu et al. (2011) showed clear activations in the *medial* orbito-frontal cortex during reward anticipation and receipt, the current meta-analysis shows that loss *anticipation* recruits instead the activity of ventro-*lateral* prefrontal regions (note: the medial PFC was actived during loss *outcome*, however). Compared to the medial OFC, which is involved in the subjective valuation of reinforcers (Noonan et al., 2012), the ventro-lateral prefrontal regions have been shown to play a significant role in emotion regulation and cognitive control (Frank et al., 2014; Levy & Wagner, 2011). This differential pattern of activity means that reward *anticipation* may have greater subjective value than the *anticipation* of loss. (note: comparatively, both reward receipt and loss receipt seem to activate medial prefrontal regions). Another difference between both types of events is that both the median cingulate gyrus is activated during loss anticipation, but not during reward anticipation and receipt (Knutson & Greer, 2008; Liu et al., 2011). Although the fMRI literature has paid far less attention to the median cingulate gyrus compared to the ACC, there is growing evidence showing that this region is a hub linking incoming affective information with brain regions involved in goal-directed behavior, and that it uses information about punishment (e.g., painful stimuli) to control action motivated by aversive events (for a review and meta-analysis, see Shackman et al., 2011). In that regard, this particular result is clearly consistent with a novel interpretation of the key roles of the median cingulate gyrus. Another noteworthy difference that we observed in the current meta-analysis is that loss events (anticipation and receipt) were associated with activations in the bilateral amygdala, which was not significantly activated in the previous meta-analyses on reward processing (Knutson & Greer, 2008; Liu et al., 2011). As such, this result lends support to the notion that the amygdala would play a significant role in the processing of negative outcomes due to its well-established role as a threat detector (LeDoux, 2014; Lutz & Widmer, 2014).

The similarity of findings observed in the current meta-analysis with the results of previous meta-analyses on the neural processing of aversive emotional stimuli raises the question of the specificity of the findings reported here. As in the current meta-analysis, previous meta-analyses of neuroimaging studies on negative emotions have shown that the ACC, (anterior) insula and amygdala are consistently activated across studies, regardless of the type of emotional stimuli (Frank et al., 2014; Fusar-Poli et al., 2009; Groenewold et al., 2013). As such, these observations suggest that the loss events and aversive emotional stimuli are processed (at least in part) via common neurobiological mechanisms. Striatal activations have also been observed in previous meta-analyses of neuroimaging studies on aversive emotional stimuli (Frank et al., 2014; Groenewold et al., 2013); however, it is important to note that striatal activations are not observed in the case of every type of negative emotion (Fusar-Poli et al., 2009). This tentatively suggests that the striatum (putamen and caudate nucleus) may play a more important role in the processing of loss events than the processing of aversive emotional stimuli. Based on the current state of knowledge, the clearest difference between loss events and aversive emotional stimuli is that the parahippocampal gyrus has been consistently found to be activated in previous meta-analyses on aversive emotional stimuli (Frank et al., 2014; Fusar-Poli et al., 2009; Groenewold et al., 2013), but *not* in the current meta-analysis. As such, this latter result suggests that the neurobiological mechanisms involved in the processing of loss events and aversive emotional stimuli may not be fully overlapping. Future studies will need to formally test these assumptions in head-to-head comparison of both types of stimuli.

The current meta-analysis has some limitations that need to be acknowledged. The first limitation has to do with the number of studies included in the meta-analysis. In the current meta-analysis, we were able to retrieve a significantly larger sample of studies than in the previous one focusing on the MIDT (35 vs 12 studies) (Knutson & Greer, 2008). Still, this sample of studies is not comparable to the number of studies included in the meta-analyses on reward events, and as such, our results should not be considered reliable. Due to this clear imbalance between the number of studies on reward and punishment, we did not perform a direct comparison between both types of events. In the same vein, the finding of similar though smaller brain regions activated during loss *receipt* relative to loss *anticipation* may simply be explained by the fact that the analysis on loss *receipt* was based on a smaller sample of studies. As in several other fMRI meta-analyses (Bartra, McGuire & Kable, 2013; Costafreda et al., 2008), heterogeneity is another limitation of the current meta-analysis. However, in an effort to explain this heterogeneity, we performed sub-analyses on age, scanner magnet intensity, smoothing kernel level and TR. We found that studies including younger participants reported stronger activations in the (dorsal) ACC and median cingulate gyrus but decreased activations in the inferior frontal gyrus, which may reflect differences in self-regulation of affective responses to punishment. Also, older participants had increased activations in the posterior cingulate cortex, a core region of the default mode network (Lin et al., 2016), which may mean that older individuals are better able to anticipate the *personal* implications of loss. Studies performed on a 1.5 Tesla scanner produced activations in the left inferior frontal gyrus, a region playing a key role in emotion regulation (Frank et al., 2014); this was not the case of studies using 3 Tesla scanners. Since the majority of studies included in the meta-analysis were performed on 1.5 Tesla scanners, known to have lower signal-to-noise ratio (Parra-Robles, Cross & Santyr, 2005), it could explain why activations in the left inferior frontal gyrus were not observed in our global analysis. Also, the relationship between TR and occipital and cerebellar activations during loss anticipation suggests that studies with long TR parameters may lack statistical power to detect activations in these regions. Finally, greater activations were observed in the right striatum in studies using a smoothing level of 4 FWHM, whereas studies a smoothing level of 8 FWHM found greater activations in the thalamus and the lingual gyrus, which is consistent with the notion that smaller smoothing levels increase the chance of finding activations in small brain regions and bias the spatial localization of striatal activity (Sacchet & Knutson, 2013).

In the largest neuro-imaging meta-analysis on loss anticipation and receipt, we found that healthy participants recruit activations in brain regions, such as the ACC, anterior insula and striatum, that are involved in affective responding. Although these regions have been shown to be activated also during reward anticipation and receipt (Knutson & Greer, 2008; Liu et al., 2011), punishment seems to recruit to a greater extent ventro-lateral prefrontal regions (loss *anticipation*) and the amygdala (loss *anticipation* and *receipt*), which are involved in emotion regulation and threat detection, respectively. In the future, more neuro-imaging research is needed on the head-to-head comparison of reward and punishment events within the same sample of participants. In order to improve the ability to detect differences between both types of events, it will be relevant to perform uni- and multi-variate analyses. In recent years, several fMRI studies have shown that multivariate analyses help overcome the multiple comparisons problem inherent to mass-univariate approaches and to improve analytic accuracy (Valente et al., 2014). Importantly, it has been shown that multi-variate approaches can also be used in the case of rapid event-related designs (Mumford et al., 2012), which are typically employed in the case of the MIDT. Given that several of the regions examined in the current meta-analysis tap into neural networks identified in large datasets of resting-state functional connectivity data (Gu et al., 2010; Seeley et al., 2007), such as the mesolimbic and salience networks, future studies will need to not only examine the *activity* of the brain regions involved in punishment processing, but also the functional and effective *connectivity* between them. Future studies will also need to pay greater attention to the differences in neural activity between loss *anticipation* and loss *receipt*, which seem to mostly differ in terms of prefrontal activations (ventro-lateral versus medial, respectively). Future studies will also need to study the neurobiological bases of the altered responses to punishment seen in some psychiatric disorders, starting with populations having high levels of harm avoidance (e.g., anxiety disorders) and those displaying, on the contrary, a relative insensitivity to punishment (e.g., psychopathy/callous-unemotional traits).

Finally, it is noteworthy to mention that the most variants of the MIDT are designed in such fashion that participants win money over the entire task. This means the most variants of the task have slightly more power to detect consistent activations during reward than loss outcomes. In the present meta-analysis, a large majority of studies used the MID task with a probability of successful trial of ≥65% (*n* = 28, 80%). As pointed out by Dillon et al. (2008) and Ubl et al. (2015), this type of design could have led to possible under-estimations of the loss-related effects (i.e., significantly more successful trial than losses). In fact, striatal and medial frontal regions were found to be maximally responsive when rewards were unpredictable (i.e., probability of successful trial at 50%) (Berns et al., 2001). Therefore, using a more unpredictable variant of the original MIDT could also be a good alternative to increase the sensitivity of mapping loss processing. Even if the results of the current meta-analysis were relatively robust, it would be of interest to use other variants of the MIDT, in the future, that are more optimized for studying loss events in terms of power and instructions. For instance, Hahn et al. (2010) used a modified version of the MIDT in which the participants started with 10 euros and were instructed to lose as little as possible. This modified version of the task could represent a good alternative to recruit more negative salience activations during loss events.

## Conclusion

To our knowledge, this is the first sufficiently powered meta-analysis to be performed on the neural mechanisms involved in both loss anticipation *and* receipt. The results of the meta-analysis provide insights on the regions that are commonly activated by reward and punishment events, as well as the regions that are potentially specific to each event type. The meta-analysis also provide a map of the brain regions that are activated during loss events that can be used a regions-of-interest for future neuro-imaging investigations on the neurobiology of psychiatric disorders characterized by harm avoidance or by an insensitivity to punishment.

##  Supplemental Information

10.7717/peerj.4749/supp-1 Supplemental Information 1 Supplementary tables and figure

10.7717/peerj.4749/supp-2 Supplemental Information 2 Rationale and contributions of the meta-analysis

10.7717/peerj.4749/supp-3 Supplemental Information 3 PRISMA checklist

10.7717/peerj.4749/supp-4 Supplemental Information 4 Raw data

## Additional Information and Declarations

### Data Availability

The following information was supplied regarding data availability: This research was a meta-analysis, therefore this article did not generate any data or code. Any data used were collected from the literature, and are provided in the Supplemental Files.
