# Supplementary material for: Loss anticipation and outcome during the Monetary Incentive Delay Task: a neuroimaging systematic review and meta-analysis
Source: PeerJ. 2018 May 10;6:e4749. doi: 10.7717/peerj.4749 (PMC5949205; doi:10.7717/peerj.4749)
Supplement: Supplemental Information 1 [file peerj-06-4749-s001.pdf]

Supplementary Table 1: PRISMA CHECKLIST

Section/topic	#	Checklist item	Reported on page #
**TITLE**			
Title	1	Identify the report as a systematic review, meta-analysis, or both.	1
**ABSTRACT**			
Structured summary	2	Provide a structured summary including, as applicable: background; objectives; data sources; study eligibility criteria, participants, and interventions; study appraisal and synthesis methods; results; limitations; conclusions and implications of key findings; systematic review registration number.	1
**INTRODUCTION**			
Rationale	3	Describe the rationale for the review in the context of what is already known.	1-2-3
Objectives	4	Provide an explicit statement of questions being addressed with reference to participants, interventions, comparisons, outcomes, and study design (PICOS).	2-3
**METHODS**			
Protocol and registration	5	Indicate if a review protocol exists, if and where it can be accessed (e.g., Web address), and, if available, provide registration information including registration number.	NA
Eligibility criteria	6	Specify study characteristics (e.g., PICOS, length of follow-up) and report characteristics (e.g., years considered, language, publication status) used as criteria for eligibility, giving rationale.	3
Information sources	7	Describe all information sources (e.g., databases with dates of coverage, contact with study authors to identify additional studies) in the search and date last searched.	3
Search	8	Present full electronic search strategy for at least one database, including any limits used, such that it could be repeated.	3
Study selection	9	State the process for selecting studies (i.e., screening, eligibility, included in systematic review, and, if applicable, included in the meta-analysis).	3
Data collection process	10	Describe method of data extraction from reports (e.g., piloted forms, independently, in duplicate) and any processes for obtaining and confirming data from investigators.	3
Data items	11	List and define all variables for which data were sought (e.g., PICOS, funding sources) and any assumptions and simplifications made.	3

Risk of bias in individual studies	12	Describe methods used for assessing risk of bias of individual studies (including specification of whether this was done at the study or outcome level), and how this information is to be used in any data synthesis.	3-4
Summary measures	13	State the principal summary measures (e.g., risk ratio, difference in means).	3-4
Synthesis of results	14	Describe the methods of handling data and combining results of studies, if done, including measures of consistency (e.g., I^2) for each meta-analysis.	3-4

Section/topic	#	Checklist item	Reported on page #
Risk of bias across studies	15	Specify any assessment of risk of bias that may affect the cumulative evidence (e.g., publication bias, selective reporting within studies).	4-5
Additional analyses	16	Describe methods of additional analyses (e.g., sensitivity or subgroup analyses, meta-regression), if done, indicating which were pre-specified.	4-5
**RESULTS**			
Study selection	17	Give numbers of studies screened, assessed for eligibility, and included in the review, with reasons for exclusions at each stage, ideally with a flow diagram.	7-8-9
Study characteristics	18	For each study, present characteristics for which data were extracted (e.g., study size, PICOS, follow-up period) and provide the citations.	6
Risk of bias within studies	19	Present data on risk of bias of each study and, if available, any outcome level assessment (see item 12).	NA
Results of individual studies	20	For all outcomes considered (benefits or harms), present, for each study: (a) simple summary data for each intervention group (b) effect estimates and confidence intervals, ideally with a forest plot.	7-8-9-10
Synthesis of results	21	Present results of each meta-analysis done, including confidence intervals and measures of consistency.	7-8-9-10, Supp T2 to T6
Risk of bias across studies	22	Present results of any assessment of risk of bias across studies (see Item 15).	8-9, Supp T2, Supp T6
Additional analysis	23	Give results of additional analyses, if done (e.g., sensitivity or subgroup analyses, meta-regression [see Item 16]).	Supp T2 to T6
**DISCUSSION**			
Summary of evidence	24	Summarize the main findings including the strength of evidence for each main outcome; consider their	10-11

		relevance to key groups (e.g., healthcare providers, users, and policy makers).	
Limitations	25	Discuss limitations at study and outcome level (e.g., risk of bias), and at review-level (e.g., incomplete retrieval of identified research, reporting bias).	12
Conclusions	26	Provide a general interpretation of the results in the context of other evidence, and implications for future research.	12
**FUNDING**			
Funding	27	Describe sources of funding for the systematic review and other support (e.g., supply of data); role of funders for the systematic review.	NA

From: Moher D, Liberati A, Tetzlaff J, Altman DG, The PRISMA Group (2009). Preferred Reporting Items for Systematic Reviews and Meta-Analyses: The PRISMA Statement. PLoS Med 6(7): e1000097. doi:10.1371/journal.pmed1000097

For more information, visit: **www.prisma-statement.org**.

Supplementary Table 2: Increased activations of the striato-insular cluster after exclusion of the outlier (n=34)

	SDM z-value(a)	*P* Value(b)	No. of voxels(c)	Breakdown (No. of voxels)(c)
Left Caudate nucleus	7.114	~0	10030	Bilateral Anterior thalamic projections (1253)
				Bilateral Insula (1234)
				Bilateral Striatum (1231)
				Bilateral BA 48 (811)
				Bilateral Putamen (723)
				Bilateral Caudate Nucleus (575)
				Bilateral Thalamus (565)
				Bilateral BA 25 (328)
				Bilateral BA 47 (315)
				Corpus callosum (225)
				Bilateral BA 45 (221)
				Bilateral BA 11 (203)
				Bilateral amygdala (121)
				Bilateral BA 34 (101)
				Anterior commissure (104)

Note. BA = Brodmann Area; SDM = Seed-based d Mapping;
(a) Voxel probability threshold: p = 0.005
(b) Peak height threshold: z = 1
(c) Cluster extent threshold: 100 voxels. Regions with less than 10 voxels are not reported in the cluster breakdown.

Supplementary Table 3. Increased activations during anticipation of monetary loss: robustness analyses

	Increased activations during loss anticipation							
	Left lenticular nucleus (Pallidum)	Left median cingulate / paracingulate gyri	Left precentral gyrus	Left Cereb Hemis. lobule VI	Right Lingual Gyrus	Right Cereb., Hemis. lobule VI	Left lingual gyrus	Right middle frontal gyrus
<u>Jackknife analysis</u>								
All studies but Balodis *et al.*	Yes	Yes	Yes	Yes	Yes	Yes	Yes	Yes
All studies but Bayer *et al.*	Yes	Yes	Yes	Yes	Yes	Yes	Yes	Yes
All studies but Beck *et al.*	Yes	Yes	Yes	Yes	Yes	Yes	No	Yes
All studies but Bjork *et al. 2004*	Yes	Yes	Yes	Yes	Yes	Yes	Yes	Yes
All studies but Bjork *et al. 2008*	Yes	Yes	Yes	Yes	Yes	No	Yes	Yes
All studies but Bjork *et al. 2010*	Yes	Yes	Yes	Yes	No	No	No	No
All studies but Bustamante *et al.*	Yes	Yes	Yes	Yes	Yes	Yes	Yes	Yes
All studies but Cho *et al.*	Yes	Yes	Yes	Yes	Yes	Yes	Yes	No
All studies but Cooper *et al.*	Yes	Yes	Yes	Yes	Yes	Yes	Yes	Yes
All studies but Dillon *et al.*	Yes	Yes	Yes	Yes	Yes	No	Yes	Yes
All studies but Enzi *et al.*	Yes	Yes	Yes	Yes	Yes	Yes	No	No
All studies but Hahn *et al.*	Yes	Yes	Yes	Yes	Yes	No	No	No
All studies but Herbort *et al.*	Yes	Yes	Yes	Yes	Yes	No	Yes	No
All studies but Juckel *et al.2006*	Yes	Yes	Yes	Yes	Yes	Yes	No	Yes
All studies but Juckel *et al.2012*	Yes	Yes	Yes	Yes	Yes	Yes	No	Yes
All studies but Kaufmann *et al.*	Yes	Yes	Yes	Yes	Yes	Yes	No	No
All studies but Kirk *et al.*	Yes	Yes	Yes	Yes	No	No	Yes	Yes
All studies but Knutson *et al.2001*	Yes	Yes	Yes	Yes	Yes	Yes	Yes	No
All studies but Knutson *et al.2003*	Yes	Yes	Yes	Yes	Yes	No	Yes	Yes
All studies but Knutson *et al.2008*	Yes	Yes	Yes	Yes	Yes	Yes	Yes	Yes
All studies but Kocsel *et al*	Yes	Yes	Yes	Yes	Yes	Yes	Yes	Yes
All studies but Pfabigan *et al.*	Yes	Yes	Yes	Yes	Yes	No	No	No
All studies but Romanczuk *et al.*	Yes	Yes	Yes	Yes	Yes	Yes	Yes	Yes
All studies but Samanez-Larkin *et al.(1)*	Yes	Yes	Yes	Yes	Yes	Yes	Yes	Yes
All studies but Samanez-Larkin *et al.(2)*	Yes	Yes	Yes	Yes	Yes	Yes	Yes	No
All studies but Schlagenhauf *et al. 2008*	Yes	Yes	Yes	Yes	Yes	No	Yes	Yes
All studies but Schlagenhauf *et al. 2009*	Yes	Yes	Yes	Yes	Yes	Yes	Yes	Yes
All studies but Stoy *et al. 2011*	Yes	Yes	Yes	Yes	Yes	Yes	No	Yes
All studies but Stoy *et al. 2012*	Yes	Yes	Yes	Yes	Yes	Yes	Yes	Yes
All studies but Treadway *et al.*	Yes	Yes	Yes	Yes	No	No	Yes	Yes
All studies but Ubl *et al.*	Yes	Yes	Yes	Yes	Yes	No	Yes	No
All studies but Van Duin *et al*	Yes	Yes	Yes	Yes	Yes	Yes	Yes	No
All studies but Wrase *et al. 2007a*	Yes	Yes	Yes	Yes	Yes	Yes	Yes	Yes
All studies but Wrase *et al. 2007b*	Yes	Yes	Yes	Yes	Yes	Yes	Yes	Yes
All studies but Wu *et al.*	Yes	Yes	Yes	Yes	Yes	Yes	Yes	Yes
	35 out of 35	35 out of 35	35 out of 35	35 out of 35	32 out of 35	24 out of 35	26 out of 35	24 out of 35

Supplementary Table 4: Increased activations during anticipation of monetary loss: magnet intensity (tesla)

	MNI Coordinates	SDM z-value[(a)]	*P* Value[(b)]	No. of voxels[(c)]	Breakdown (No. of voxels) [(c)]
Comparisons					
3 < 1.5T scanners					
Right inferior frontal gyrus operc. Part.	42,12,12	2.238	0.00037	1062	R BA 48 (441)<br>R insula (201)<br>R BA 38 (130)<br>R BA 21 (25)<br>Corpus callosum (27)
Left inferior frontal gyrus, Opercular Part.	-50,14,16	2.068	0.00077	297	L BA 48 (103)<br>L BA 44 (85)<br>L BA 6 (51)
3 > 1.5T scanners					
Left cerebellum, hemispheric lobule VI	-28,-70,-22	2.859	0.00015	1295	L cerebellum, hemispheric lobule VI (581)<br>L cerebellum, crus I (367)<br>L fusiform gyrus (200)
Left thalamus	-10,-14,4	3.58	0.000005	350	L anterior thalamic (301)<br>L pons (19)<br>Corpus callosum (16)

Note. BA = Brodmann Area; SDM = Seed-based d Mapping; (a) Voxel probability threshold: p = 0.0005; (b) Peak height threshold: z=1; (c) Cluster extent threshold: 100 voxels. Regions with less than 10 voxels are not reported in the cluster breakdown.

Supplementary Table 5: Increased activations during anticipation of monetary loss: comparisons full width at half maximum (FWHM) of the smoothing kernel.

Comparisons	*MNI* Coordinates	SDM z-value[(a)]	*P* Value[(b)]	No. of voxels[(c)]	Breakdown (No. of voxels) [(c)]
8 < 4 mm FWHM					
Right insula	26,14,-16	3.819	~0	186	R frontal orbito-polar tract (46)<br>R BA 11 (72)<br>R Insula (16)<br>R BA 48 (14)
8 > 4 mm FWHM					
Left lingual gyrus	-6,-84,2	2.25	~0	512	L BA 17 (155)<br>Corpus callosum (98)<br>R BA 17 (63)<br>L BA 18 (56)
Left thalamus	-10,-12,8	2.14	0.000005	120	L anterior thalamic (119)

Note. BA = Brodmann Area; SDM = Seed-based d Mapping.
(a) Voxel probability threshold: p = 0.0005
(b) Peak height threshold: z = 1
(c) Cluster extent threshold: 100 voxels. Regions with less than 10 voxels are not reported in the cluster breakdown.

Supplementary Table 6: Increased activations during loss anticipation: meta-regression on age

	MNI Coordinates	SDM z-value(a)	*P* Value(b)	No. of voxels(c)	Breakdown (No. of voxels) (c)
Effects of Age					
Younger < Older					
Left inferior frontal gyrus, opercular part	-54,8,22	2.62	0.0011	190	L BA 44 (89)
					L BA 6 (65)
L Median cingulate	-8,-30,26	2.826	0.00057	182	L posterior cingulate gyrus (76)
					L median network, cingulum (53)
					Corpus callosum (45)
Younger > Older					
Left anterior cingulate/ paracingulate gyri	-8,24,30	2.247	0.00019	941	Bilateral anterior cingulate/paracingulate gyri (425)
					Bilateral median cingulate/paracingulate gyri (333)
					L superior frontal gyrus (155)
Right olfactory cortex	2,12,-4	2.748	~0	611	Bilateral striatum (199)
					Bilateral BA 25 (191)
					R anterior thalamic projections (79)
Left thalamus	-14,-24,-2	2.589	~0	550	L thalamic projections (376)
					L pons (38)
Right lingual gyrus	12,-58,-4	2.085	0.00048	182	R BA 18 (110)
					R BA 19 (44)

Note. BA = Brodmann Area; SDM = Seed-based d Mapping.
(a) Voxel probability threshold: p = 0.005
(b) Peak height threshold: z = 1
(c) Cluster extent threshold: 100 voxels. Regions with less than 10 voxels are not reported in the cluster breakdown.

Supplementary Table 7: Increased activations during anticipation of monetary loss: meta-regression on repetition time (TR)

	MNI Coordinates	SDM z-value[(a)]	*P* Value[(b)]	No. of voxels[(c)]	Breakdown (No. of voxels) [(c)]
Effects of TR					
Shortest TR < Longest TR					
-					
Shortest TR > Longest TR					
Lingual Gyrus	0,-88,4	-2.991	0.00005	461	L BA 17 (195)<br>L BA 18 (96)<br>Corpus Callosum (90)
Cerebellum, vermic lobule VI	6,-78,-16	-2.601	0.0003	297	BA 18 (168)<br>BA 17 (50)

Note. BA = Brodmann Area; SDM = Seed-Based d Mapping
(a) Voxel probability threshold: p = 0.005
(b) Peak height threshold: z = 1
(c) Cluster extent threshold: 100 voxels. Regions with less than 10 voxels are not reported in the cluster breakdown.

Supplementary Table 8. Increased activations during loss outcome: robustness analyses.

	Increased activations during monetary loss outcome		
	Right striatum	Left anterior cingulate / paracingulate gyri	Left striatum
Jackknife analysis			
All studies but Balodis *et al.*	Yes	Yes	Yes
All studies but Beck *et al.*	Yes	Yes	Yes
All studies but Bjork *et al. 2004*	Yes	Yes	Yes
All studies but Bjork *et al. 2010*	Yes	Yes	Yes
All studies but Cooper *et al.*	Yes	Yes	Yes
All studies but Dillon *et al.*	Yes	Yes	Yes
All studies but Kirk *et al.*	Yes	Yes	Yes
All studies but Knutson *et al.2008*	Yes	Yes	Yes
All studies but Kocsel *et al*	Yes	Yes	Yes
All studies but Romanczuk *et al.*	Yes	Yes	Yes
All studies but Samanez-Larkin *et al.(1)*	Yes	Yes	Yes
All studies but Samanez-Larkin *et al.(2)*	Yes	Yes	Yes
All studies but Schlagenhauf *et al. 2009*	Yes	Yes	Yes
All studies but Treadway *et al.*	Yes	Yes	Yes
All studies but Ubl *et al.*	Yes	Yes	Yes
All studies but Wu *et al.*	Yes	Yes	Yes
	16 out of 16	16 out of 16	16 out of 16

Supplementary Figure 1. Funnel plot of the striato-insular cluster in the anticipation of loss condition (n=34).

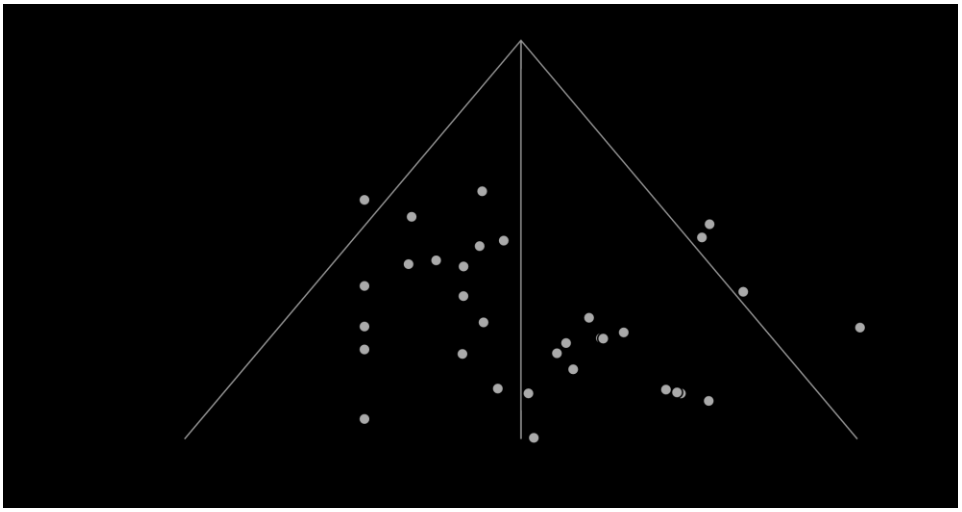

Note. SE = Standard Error.
