# Supplementary material for: Loss anticipation and outcome during the Monetary Incentive Delay Task: a neuroimaging systematic review and meta-analysis
Source: PeerJ. 2018 May 10;6:e4749. doi: 10.7717/peerj.4749 (PMC5949205; doi:10.7717/peerj.4749)
Supplement: Supplemental Information 2 [file peerj-06-4749-s002.docx]

* Justification for the meta-analysis: Reward seeking and avoidance of punishment are key motivational processes. Although the bulk of neuro-imaging research has been done on reward processing, little is known about the neural basis of punishment processing. Therefore, we conducted a meta-analysis of brain activations during anticipation and receipt of monetary losses in healthy controls, using the *Monetary Reward Delay Task*.

* Knowledge gained: The current meta-analysis highlighted activations, during loss events, in brain regions that have been shown to be activated also during reward events (e.g. medial prefrontal cortex / anterior cingulate cortex, anterior insula and striatum), as well as activations in brain regions that are potentially specific to loss events, such as the ventro-lateral prefrontal cortex, the median cingulate cortex and the amygdala.
